# Recent Advance on Polyaniline or Polypyrrole-Derived Electrocatalysts for Oxygen Reduction Reaction

**DOI:** 10.3390/polym10121397

**Published:** 2018-12-17

**Authors:** Zhankun Jiang, Jiemei Yu, Taizhong Huang, Min Sun

**Affiliations:** School of Chemistry and Chemical Engineering, University of Jinan, 336 West Nanxinzhuang Road, Jinan 250022, China; chm_yujm@ujn.edu.cn (J.Y.); chm_huangtz@ujn.edu.cn (T.H.)

**Keywords:** oxygen reduction reaction, fuel cell, polyaniline, polypyrrole, *N*-doped carbon

## Abstract

The fuel cell, as one of the most promising electrochemical devices, is sustainable, clean, and environmentally benign. The sluggish oxygen reduction reaction (ORR) is an important fuel cell cathodic reaction that decides the efficiency of the overall energy conversion. In order to improve ORR efficiency, many efficient catalysts have been developed, in which the *N*-doped material is most popular. Polyaniline and polypyrrole as common aromatic polymers containing nitrogen were widely applied in the *N*-doped material. The shape-controlled *N*-doped carbon material can be prepared from the pyrolysis of the polyaniline or polypyrrole, which is effective to catalyze the ORR. This review is focused on the recent advance of polyaniline or polypyrrole-based ORR electrocatalysts.

## 1. Introduction

A fuel cell is a device that directly transfers chemical energy stored in fuel and oxidizer to electrical energy [1,2,3]. It is different from conventional batteries in that the fuel cell generates electricity as long as the fuel is continuously supplied. Because it is not restricted by the Carnot cycle, the energy conversion efficiency can reach 40–60%, 1.5–2 times that of the internal combustion engine, it is also environmentally friendly (emissions of CO_2_ or water) and produces no noise. Therefore, it is considered to be the most promising clean and efficient power generation technology. One major hindrance of fuel cell application is the sluggish oxygen reduction reaction (ORR) [4,5,6]. The reaction occurs on the cathode as a half reaction. The commercial Pt/C catalysts are the most applied catalysts [7,8,9]. However, platinum is rare and very expensive. In addition, Pt particles may be peeled off or aggregate in the catalysis process, which causes a decrease in activity [10,11,12,13]. Therefore, the development of efficient ORR catalysts is one of the most important factors for the fuel cell.

In order to replace Pt/C, three types of electrocatalysts are mainly researched, including non-metal catalysts [14,15], non-noble metal catalysts [16,17,18,19], and low-content noble metal catalysts [20,21]. Carbon materials, as the essential support of electrocatalysts, were usually doped by a heteroatom (N, B, P, S, etc.) [22,23,24,25,26] to improve the activity of ORR electrocatalysts. The nitrogen-doped carbons are the most promising catalyst materials for ORR [27,28,29]. Two types of aromatic polymers including polyaniline (PANI) and polypyrrole (PPy) are used to provide nitrogen atoms and control the morphology.

PANI and PPy are both widely used conductive polymers [30,31,32]. Their morphology can be controlled with different synthesis methods. After pyrolysis, the morphology-controlled nitrogen doping carbon can be obtained. Furthermore, the nitrogen content of the polymer derived *N*-doped carbon is also controllable. This mini-review focuses on PPy- and PANI-derived electrocatalysts for the oxygen reduction reaction (ORR). Because the two main challenges in the ORR catalysts design are high efficiency and stability, many PANI- and PPy-derived materials have been designed. This review presents the representative works and suggests potential prospective researches.

## 2. Polyaniline-Derived Catalysts for Oxygen Reduction Reaction

In order to get efficient ORR catalysts, metal-free materials, noble metal-free materials, and noble metal materials are normally used. The noble metal sticks to the Pt and Pt alloys. Polyaniline-derived catalysts are efficient for ORR.

### 2.1. Metal-Free Polyaniline-Based Catalysts

*N*-doped carbon, which is one of the most promising ORR catalysts, can be obtained through the pyrolysis of PANI-based materials. The effect of nitrogen is still under study. The N species contain pyridinic-, pyrrolic-, and graphitic-nitrogen. Pyridinic N possesses better activity than graphitic N because of their different sp^2^ electronic structures [33]. For the metal-free PANI-based ORR catalysts, researchers make many efforts to control the morphology of the catalysts. The PANI nanotubes were pyrolyzed at different temperatures. After pyrolysis, the morphology of the nanotubes was maintained and typical nitrogen species, such as pyrrolic-, pyridinic-, and graphitic-N, were obtained. The product fabricated at 700 °C (NCNT-700) exhibited the highest electrocatalytic ORR activity, long-standing stability, and good tolerance against methanol. The half-wave potential of the NCNT-700 is 0.84 V vs. RHE. The improved activity is mainly attributed to the high nitrogen level of the active pyridinic and graphitic N. JingJing Xu et al. [34] developed a highly efficient ORR catalyst derived from PANI@CNTs-sulfonated polystyrene. Quilez-Bermejo Javier et al. [35] studied the activity of N-doped carbons derived from PANI. When the pyrolysis was processed at a high temperature above 1100 °C, the conversion from pyridine to quaternary N in the edge position occurred and resulted in excellent ORR activity. The catalysts derived from PANI nanofiber and glucose showed high onset potential (−0.171 V vs. Ag/AgCl), large limiting current density, and a 4-electron process [36]. Perchloric acid (HClO_4_) was used as an oxidant and pore-forming agent in an electrochemical polymerization [37]. In the first step, carbon paper was used in a traditional three-electrode system. HClO_4_ and aniline were used as the electrolyte solution. Secondly, the materials were carbonized under a nitrogen atmosphere. The obtained material shows a high surface area of 1341.12 m^2^ g^−1^ and high N content. PANI-derived mesoporous carbon was obtained based on yolk-shell nanostructured polyaniline@SiO_2_ particles [38], and the SiO_2_ particles were used as the hard template. The electrocatalyst presented high stability and tolerance to CH_3_OH. B/N [39] and N,P [40] co-doped carbon materials were developed and showed high ORR activity. Quilez-Bermejo Javier et al. [41] heated the PANI and de-doped PANI (PANId) under two different atmospheres: a pure inert atmosphere (N_2_) and a slightly oxidizing mixture of gases (3000 ppm O_2_ in N_2_). Interestingly, the pyrolysis under 800 °C using a slight oxidant atmosphere produces carbon materials with much higher ORR activity. The authors believe the larger amount of N-edge and O-edge sites contribute to the phenomenon. Therefore, in the pyrolysis process, the commonly used inert atmosphere may not the best choice.

### 2.2. Noble Metal-Free Polyaniline-Based Catalysts

Some nonprecious metals, represented by iron, cobalt, nickel, and manganese, were used to generate the efficient ORR electrocatalysts. Among the non-noble metal-based ORR catalysts, Fe-N-C is one of the most promising materials [42,43,44,45]. Compared to commercial Pt/C, Fe-N-C has several advantages such as low price, high efficiency, tolerance to the toxicity of CO, and long life.

Gang Wu et al. [46] incorporated iron and/or cobalt in the PANI-derived carbon catalyst. Firstly, aniline oligomer was mixed with carbon particles and transition metal precursors (cobalt nitrate and/or iron chloride), followed by the addition of ammonium persulfate as an oxidant to fully polymerize the aniline. After the polymerization process, the materials underwent pyrolysis in an N_2_ atmosphere. Among the prepared materials, PANI-Fe-C and PANI-FeCo-C materials showed similar ORR activity, and the half potential of the materials was slightly lower than that of Pt/C. The mechanism study showed a four-electron process, and the hydrogen peroxide yield was smaller than 1.0%. The PANI-derived catalysts showed high durability according to the fuel-cell performance test as well as the RDE test.

In order to insight the active sites of Fe-N-C catalyst, PANI and cyanamide were used as nitrogen precursors [16]. The prepared materials showed much larger micropore surface area and the volumes of mesopores (pore diameter 2 to 50 nm) was ~0.25 cm^3^ g^−1^. The authors concluded that the edge-hosted FeN_4_ sites contribute to the high activity in the Fe-N-C catalyst. Researchers found that a pore width between 5 and 20 angstroms [47] had a great influence on activity. The prepared material has a large mesopore surface area. When the precursor is PANI alone, the Brunauer–Emmett–Teller (BET) surface area is nearly 1000 m^2^ g^−1^, and when the precursor is PANI and CM, the surface area is nearly 1600 m^2^ g^−1^. The prepared catalysts processed a four-electron pathway, and the yield of hydrogen peroxide was lower than 2%. Carbon-embedded nitrogen-coordinated iron (FeN_4_) was proposed as the catalytic active site. Aberration-corrected scanning transmission electron microscopy was used to visualize the FeN_4_ site, and the contributions of these active sites associated with specific lattice-level carbon structures were explored computationally.

**Figure 1:**
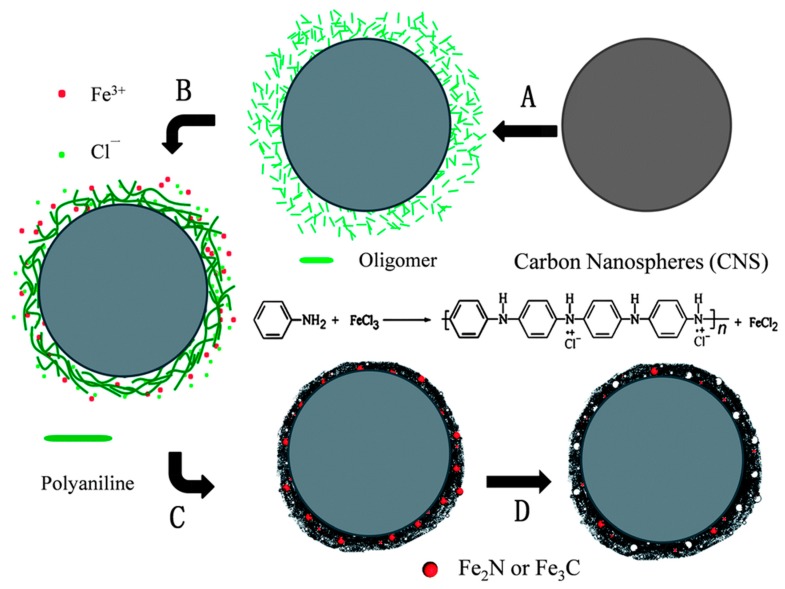
Schematic of the synthesis process of the FeN_x_C/C–F catalyst [51]. (**A**) Mixing of CNS with aniline oligomers. (**B**) Oxidative polymerization of aniline by addition of FeCl_3_ solution. (**C**) First heat treatment in an ammonia atmosphere. (**D**) Acid leaching. The second heat treatment after acid leaching is not shown.

Yang Hu et al. [48] used PANI nanofibers as nitrogen and carbon precursors. The mass content of Fe was 0, 0.3, 0.5, 1.0, 3.0, 5.0, and 10.0 wt %. The 3.0 Fe-PANI catalyst showed the best onset potential: 0.905 V vs. RHE. The materials process a four-electron ORR pathway. Prussian blue analogue (PBA, Co_3_[Fe(CN)_6_]_2_) and polyaniline (PANI) were mixed as the precursor [49]. The 2–5 nm PBA nanocrystals homogeneously dispersed in PANI. The PBA nanocrystals are the precursor for the active sites, and are also the template for pore formation in the pyrolysis process. The catalysts exhibit ORR activity comparable to that of the commercial Pt/C (20 wt % Pt loading) in the alkaline and acidic environment. Carbon nanotubes (CNTs) were used to support Fe-PANI [50]. Jian Zhang et al. [51] pyrolyzed the polyaniline on carbon nanospheres, and ferric chloride was used as an oxidant and iron source. The scheme is shown in Figure 1. The authors concluded that the as-prepared catalyst shows a high activity and much better stability than that of commercial Pt/C in an acid medium. Guanghua Wang et al. [52] prepared an N-doped carbon catalyst with trace iron (0.54 wt. % Fe). The PANI-iron coordination polymers were pyrolyzed.

Other nonprecious metals, such as cobalt, nickel, and manganese, were also used to generate electrocatalysts for ORR. Shiyi Cao et al. [53] synthesized mesoporous hybrid shells of carbonized polyaniline (C-PANI)/Mn_2_O_3_. The manganese oxide hybrid materials showed high ORR electrocatalytic activity. In particular, the onset potential is +0.974 V (versus RHE), the specific current is 60.8 mA/mg, and the electronic transfer number is 4. The remarkably high ORR activity can be attributed to the high specific surface area, the surface oxidation state of Mn, and composition-codependent behavior. PANI and beta-MnO_2_ nanocomposites [54] were also built. PANI nanofibers were hybridized with cobalt nitrate [55]. After pyrolysis, the PANI nanofibers formed graphene networks with *N*-doped. It is a facile and scalable approach to the synthesis of Co and N codoped graphene networks for ORR in acidic solutions with high activity and excellent durability. Furthermore, the NiCo-doped PANI precursors underwent pyrolysis in an inert atmosphere at 800 °C [56], and the molar ratio of Ni and Co was adjusted. Among the prepared catalysts, the catalyst Ni_6_Co_1_/C-N, which showed the largest surface area according to the BET method, presented the best ORR activity and stability. PANI was used to reduce Ag^+^ cations [57] to get catalysts. The Ag dendrites were readily generated in PANI nanofiber dispersion via the redox reaction between AgNO_3_ and PANI.

### 2.3. Noble Metal Polyaniline-Based Catalysts

In the harsh chemical and electrochemical conditions, the carbon supports are susceptible to corrosion. In order to increase the stability, which is the key factor in catalyst application, PANI was used as a protector to inhibit carbon nanospheres from corrosion of the carbon supports. In order to improve the durability of the Pt-based ORR catalysts, perfluorosulfonic acid (PFSA) and PANI were used to co-stabilize Pt catalysts [58]. The prepared Pt-PFSA/C@PANI catalyst shows comparable activity with the commercial Pt/C. Furthermore, the catalyst shows much higher stability than the commercial Pt/C. The stability is very important for this Pt-based catalyst. The stability can be concluded to the result of PFSA and PANI. The Pt NPs was wrapped by PFSA (Pt@PFSA). Then the Pt@PFSA were anchored on C@PANI. The coating of PANI on carbon supports can cover the surface of the carbon supports, and this causes the micropores on the surface of the carbon to disappear. The phenomenon can prevent Pt NPs being embedded in the micropores. The dual PFSA and PANI polymers are important for the stability of the as-prepared catalyst.

The Pt-based alloy is also used to enhance the ORR activity and reduce the Pt content [59]. Yang Liu et al. [60] prepared several Pt-Co/C-PANI catalysts via a microwave-assisted polyol method. The best-prepared catalysts showed a mass activity of 1.33 A mg_Pt_^−1^ and specific activity of 1.29 mA cm^−2^, and the performance was 7.8 and 5.4 times higher than that of Pt/C catalyst. The ORR activity and stability of Pt and PtM (M = Ni, Co, Cr, Pd) supported on polyaniline/CNTs were explored and compared with the commercial Pt/C catalyst [61].

## 3. Polypyrrole-Derived Catalysts for Oxygen Reduction Reaction

Like the PANI-derived catalysts, the polypyrrole-derived catalysts can also be divided into 3 kinds: metal-free, noble metal-free, and noble metal.

### 3.1. Metal-Free Polypyrrole-Derived Catalysts

**Figure 2:**
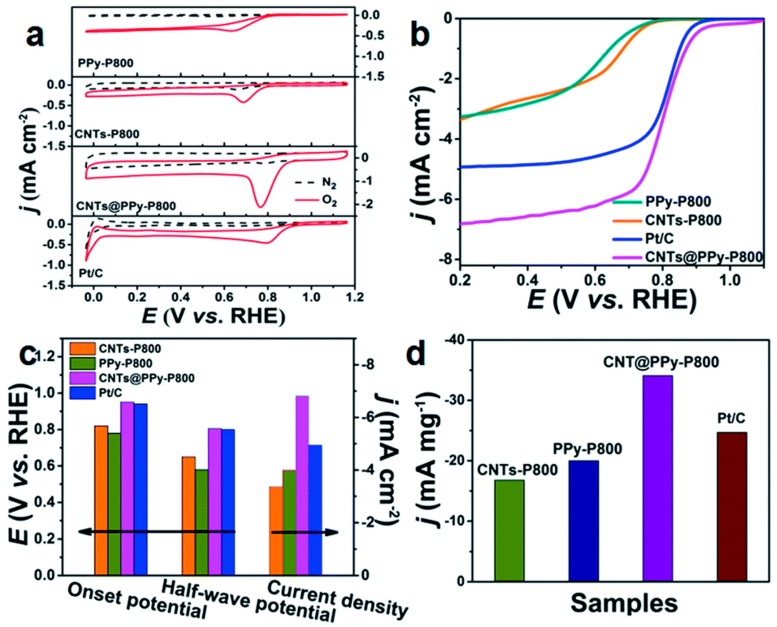
(**a**) CV curves of PPy-P800, CNTs-P800, CNTs@PPy-P800, and Pt/C in N_2_ and O_2_-saturated solution, respectively. (**b**) LSV curves of PPy-P800, CNTs-P800, CNTs@PPy-P800, and Pt/C catalyst in O_2_-saturated 0.1 M KOH solution at a sweep rate of 10 mV s^−1^ and an electrode rotation speed of 1600 rpm. (**c**) The onset potential, half-wave potential, and current density of PPy-P800, CNTs-P800, CNTs@PPy-P800, and Pt/C, respectively. (**d**) Corresponding mass activities of these four samples [62].

Hongli An et al. [62] polymerized PPy in a CNT matrix, then the CNTs were covered with PPy on the surface (CNTs@PPy). Then the hybrid material underwent pyrolysis to obtain an N-doped CNT. The ORR activity is shown in Figure 2. The as-obtained NCNTs exhibited an onset potential of 0.95 V, a diffusion-limited current of 6.82 mA cm^−2^, and excellent stability in alkaline media. The results indicate that the high ORR performances were mainly derived from the pyridinic-N in the NCNTs. Furthermore, the ratio of three different nitrogens (pyridinic, pyrrolic, or graphitic N) can be easily tuned by adjusting the amount of PPy in the CNTs@PPy core-shell precursors.

In order to improve the activity and lifetime of the catalysts, other heteroatoms such as P, S, and F were also added. Phytic acid was used as P-dopant, and polystyrene sphere was used as a template [63]. After the pyrolysis process, the resultant N,P-MC materials exhibited spherical mesopores with an average diameter of ca. 70 nm. The BET surface area of the N,P-MC sample is 305 m^2^ g^−1^. N,P-MC exhibits best ORR activity among the prepared materials. The material performs a 4-electron process, has high ORR activity, and good methanol tolerance. The mesoporous structure, high surface area, and increased active sites (N,P) are key factors that improve ORR performance. Researchers [64] synthesized nitrogen-doped carbon nanofiber films (NCNFs) via the carbonization of polypyrrole-functionalized electrospun polyacrylonitrile (PAN) nanofibers. A triazine-based polypyrrole network (TPN) was synthesized and then pyrolyzed to get an N-rich carbon catalyst [65]. In the TPN synthesis process, the 2,4,6-tripyrrol-1,3,5-triazine monomer was used; the protonating agent was TfOH and the oxidizing agent was benzoyl peroxide. The obtained NC-900 (pyrolyzed at 900 °C) catalyst, which presents a surface area of 779 m^2^ g^−1^ and contains 3.02% nitrogen, exhibits promising ORR activity in alkaline media. Two key ORR parameters—onset potential and half-wave potential of prepared NC-900—are both higher than those of Pt/c. Furthermore, the as-prepared material shows better MeOH tolerance and higher durability.

The relationship between the morphology of PPy and the corresponding ORR activity were studied [66]. The granular- and tubules-like PPy was annealed at 800 °C with morphology maintained. For the ORR catalyst performance, such as onset potential, half-wave potential, electron number, and stability, the tubules-like carbon was better than the granular-like carbon. The higher catalytic activity was explained by a better electrical conductivity in the tubular structure than in the granular one.

### 3.2. Non-Noble Metal Polypyrrole-Derived Catalysts

A series of Fe-N-C electrocatalysts derived from PPy were designed and studied [67]. In the catalysis preparation, mesoporous carbon (MPC), PPy, and Fe(II) acetate were used as the C source, N source, and Fe source, respectively. Firstly, polyvinylpyrrolidone (PVP) was added to a MPC-PPy support, and the hybrid material was heat-treated. Secondly, the pyrolysis carbon support was further impregnated with Fe^2+^ ions. The authors concluded that the microporosity of the prepared catalysts directly influences the ORR activity. The same research team [68] synthesized a Fe-Co-N-C catalyst for ORR according to a sacrificial method. Also, pyrrole was used as an inexpensive precursor for N-doped carbon materials. Alkaline membrane fuel cells (AMFC) were used in the research. A high performance for AMFC, 420 mW cm^−2^ at 60 °C, was achieved. Furthermore, a good performance of alkaline direct ethanol fuel cells was achieved. Min Sun et al. [69] use electrochemical polymerization method to synthesize structured Fe-N-C ANFs/CP (activated NFs derived from PPy doped with iron atoms in situ grown on carbon paper) on a carbon paper. The authors focused on the connection between iron concentration and ORR activity. The research showed that the 0.05-Fe-N-C ANFs exhibited the highest activity and good durability. The high activity is ascribed to the high Fe-N_x_ concentration, the porous structure, and well-dispersed active sites.

A novel kind of tetrazine-based polypyrrole spheres (PTPys) was prepared by protonic acid catalyzed Friedel–Crafts polymerization of bis(*N*-pyrrolyl)-1,2,4,5-tetrazine (TPy) with dimethoxymethane in dichloroethane [70]. Firstly, PTPys with a diameter of 100 to 300 nm were synthesized and pyrolyzed at 900 °C to generate N-doped porous carbon spheres (N/Cs-900 electrocatalyst). Then Fe(OAc)_2_ were mixed with PTPys and then pyrolysis to get N/Fe-codoped porous carbon spheres (Fe/N-Cs-900 electrocatalyst). The Fe/N-Cs-900 catalyst showed the best ORR activities. In the acidic solution, the Fe/N-Cs-900 catalyst exhibited excellent activity, which is comparable to the Pt/C catalyst. In the alkaline solution, the Fe/N-Cs-900 catalyst showed better ORR performance than Pt/C. The well-defined spherical architecture, high N content, high surface area, and porosity contributed to the high ORR activities. Haodong Sha et al. [71] experimentally studied the active sites for ORR catalysts. A series of carbon-supported cobalt-polypyrrole-4-toluenesulfinic acids were pyrolyzed in an inert atmosphere at 800 °C, then electrochemically evaluated in aqueous 0.5 M sulfuric acid. A series of catalysts were prepared. Metallic cobalt, cobalt oxide, and nitrogen species (Co-N_x_) bonded to cobalt were formed. Co-N_x_, which is the active site, was formed when the cobalt loading was less than 1.0 wt %. When the loading was higher than 1.0 wt %, metallic Co and Co oxide particles coexisted with the Co-N_x_ compound. Both metallic Co and Co oxide were not active for catalyzing ORR. As a result, at a Co loading of ~1.0 wt %, the catalyst gave the best ORR activity. The ORR active site in (Co-PPy-TsOH/C)_P_ catalyst was likely a Co-pyridinic-N group. A ternary CoNiMn-layered double hydroxide (LDH)/PPy/reduced graphene oxide (rGO) composite was fabricated by one-step involving the formation of the LDH and polymerization of the pyrrole (Py) [72].

Non-noble metal oxides were also studied. A novel strategy based on block copolymer self-assembly in solution were developed recently [73]. The prepared materials were two-dimensional graphene-based mesoporous nanohybrids with well-defined large pores of tunable sizes. In the process, polystyrene-block-poly (ethylene oxide) spherical micelles were used as the pore-creating template. The PPy monolayers were grown on both sides of rGO nanosheets, and Fe_2_O_3_ NPs were embedded in them (denoted as mPPy-Fe_2_O_3_@rGO). Furthermore, the materials were heated at 800 °C to get sandwich-like mesoporous nitrogen-doped carbon/Fe_3_O_4_/rGO (mNC-Fe_3_O_4_@rGO). The prepared mNC-Fe_3_O_4_@rGO materials show excellent electrocatalytic activity with a four-electron transfer nature, a high half-wave-potential of +0.84 V, and a limiting current density of 5.7 mA cm^−2^. Co_3_O_4_ nanoparticles were assembled on a polypyrrole/graphene oxide electrocatalyst ((CoO_4_)-O-3/Ppy/GO) as an efficient catalyst for the oxygen reduction reaction (ORR) in alkaline media [74]. By one-step in situ ball milling of graphite, pyrrole, and cobalt salt without resorting to high-temperature annealing, a facile strategy was developed to synthesize cobalt oxide and PPy coupled with a graphene nanosheet (Co_3_O_4_-PPy/GN) complex [75]. The as-prepared Co_3_O_4_-PPy/GN catalysts showed excellent electrocatalytic performances for ORRs.

Ag nanoparticles were loaded on oxygen-doped carbonaceous polypyrrole nanotubes (OCPN) [76] The Ag/OCPN catalysts possess comparable excellent activity with commercial Pt/C in alkaline solution. Moreover, the prepared catalysts showed superior stability and methanol tolerance.

### 3.3. Noble Metal Polypyrrole-Based Catalysts

In order to reduce the consumption of Pt material, Pt combined with other materials were widely investigated. Polypyrrole-based materials were widely used. Dendritic PtCo nanoclusters supported on sheet-like PPy (PtCo NCs/PPy) was proposed [77]. The synthesis method is a facile one-pot solvothermal method. Cetyltrimethylammonium chloride and pyrrole were applied as the capping agent and reductant, respectively. Meanwhile, under solvothermal conditions, the pyrrole was in situ polymerized to form PPy sheets. The prepared dendritic PtCo NCs/PPy showed an enlarged electrochemically active surface area (EASA, 30.95 m^2^ g^−1^). As compared with Pt_1_Co_3_ NPs, Pt_3_Co_1_ NPs, and commercial Pt/C catalysts, PtCo NCs/PPy showed the best catalytic performance and durability. The PPy sheets, as the supporting nitrogen-rich carbon materials, are essential in the material. Furthermore, PPy was used to modify mesoporous carbon black as the supporting material [78]. Ru-Pt NPs (1–2 nm) were dispersed on the PPy-modified carbon. In the process of PPy-modified carbon preparation, the amount of pyrrole was investigated. In order to test the ORR activities and the methanol tolerance of the material, the prepared electrocatalysts were tested according to both conventional electrochemical techniques and a direct methanol single cell. It is inspiring that the ORR performance of Ru-Pt/C-PP was far superior to that of Pt/C in the direct methanol fuel cell. The summary of electrocatalysts for ORR is shown in Table 1 and Table 2.

## 4. Summary and Perspective

Polymer-modified electrocatalysts are very promising materials for ORR. Polymers contain nitrogen atoms, which represented by PANI and PPy have been studied thoroughly. PANI or PPy alone or combined with carbon-based materials (nanoparticles, nanotubes, graphene, etc.) are pyrolyzed to generate N-doped carbon. Furthermore, different metals such as platinum, iron, cobalt, manganese, nickel, etc. are used to modify the N-doped carbon. As Table 1 shows, when binary metals are combined, such as Fe combined with Co, better ORR performance is achieved than when using single Fe or Co alone.

The ORR activities under different element additions are very interesting. Different elements, such as S, B, P, Fe, Co, Ni, Pd, Pt, etc., can be added to the catalysts. When, how, and how many of the elements are added to the catalysts are very important. When more than one metal element is added, normally we get several kinds of alloy nanoparticles, which are the important active sites. In a word, the additional elements matter in ORR catalysts.

For ORR catalysts, the pore area and size are very important. Having a pore size from 0.5 to 200 nm is very important in ORR activities. Further researches are needed to identify the best pore size. The phenomenon indicates that the polymers-derived materials should pay more attention to the pore structure and size.

In order to understand the active sites of the ORR catalysts, especially the nitrogen active sites, nitrogen-containing polymers could be designed for this purpose. Because there are many types of polymers with different nitrogen structure, they can be used as precursors of the catalysts. Furthermore, there are different morphologies of polymers, such as nanospheres, nanorods, microspheres, fibers, 3D structure gel, etc. These structures are interesting as precursors, and the relationship between activities and morphologies requires further research.

## Figures and Tables

**Table 1:** Summary of noble metal-free electrocatalysts for oxygen reduction reaction (ORR).

Electrocatalyst	Medium	Catalysts Loading [mg cm^−^^2^]	Rotation Speed/rpm	Onset Potential/V vs. RHE	Halfwave Potential/V vs. RHE	Ref.
PANI-FeCo-C	0.5 M H_2_SO_4_		900	0.93	0.79	[46]
3.0 Fe-PANI-L	0.5 M H_2_SO_4_	0.6	1000	0.905	-	[48]
C-2PANI/PBA	0.5 M H_2_SO_4_	0.36	1600	0.81	0.71	[49]
FeNxC/C–F	0.1 M HClO_4_	0.8	1600	0.88	0.76	[51]
N/C/Fe-c	0.5 M H_2_SO_4_	0.48	900	0.78	0.65	[52]
Fe/N-Cs-900	0.5 M H_2_SO_4_	-	1600	0.845	0.717	[70]
(Co-PPy-TsOH/C)P-A-P	0.5 M H_2_SO_4_		900	0.78	0.71	[71]
CPANI/Mn_2_O_3_	0.1 M KOH	0.28	1600	0.83	0.68	[53]
Fe-Co-N-C	0.1 M KOH	0.635	800	0.85	0.78	[68]
0.1-Fe-N-C ANFs	0.1 M KOH	-	1600	0.83	0.74	[69]
mNC-Fe_3_O_4_@rGO-2	0.1 M KOH	0.24	1600	0.96	0.83	[73]

**Table 2:** Summary of metal-free electrocatalysts for ORR.

Electrocatalyst	Medium	Catalysts Loading [mg cm^−^^2^]	Rotation Speed/rpm	Onset Potential/V vs. RHE	Halfwave Potential/V vs. RHE	Ref.
NCNT-700	1 M NaOH	0.4	1600	0.88 at RRDE	0.8 V at RRDE	[33]
NCNTs-900	0.1 M KOH	0.64	1600	0.96	0.82	[34]
MEP-NC850	0.1 M KOH	0.25	1600	0.94	0.82	[37]
CNTs@PPy-P800	0.1 M KOH	0.2	1600	0.92	0.81	[59]
N,P-MC	0.1 M KOH	0.2	1600	0.93	0.84	[63]
NCNFs-900	0.1 M KOH		1600	0.92	0.82	[64]
NC-900	0.1 M KOH		1600	0.93	0.84	[65]
